# Ticks and Tick-borne diseases in Ireland

**DOI:** 10.1186/s13620-017-0084-y

**Published:** 2017-01-31

**Authors:** Annetta Zintl, Sara Moutailler, Peter Stuart, Linda Paredis, Justine Dutraive, Estelle Gonzalez, Jack O’Connor, Elodie Devillers, Barbara Good, Colm OMuireagain, Theo De Waal, Fergal Morris, Jeremy Gray

**Affiliations:** 1 UCD School of Veterinary Medicine, University College Dublin, Belfield, Ireland; 2 UMR BIPAR, ANSES, INRA, ENVA, Animal Health Laboratory, 14 rue Pierre et Marie Curie, 94700 Maisons-Alfort, France; 3 Department of Zoology, Trinity College Dublin, Dublin 2, Ireland; 4 Ecole Nationale Veterinaire de Toulouse, Toulouse, France; 5 Paul Sabatier University, Toulouse, France; 6 MSD Animal Health, Dublin, Ireland; 7 Animal & Grassland Research and Innovation Centre, Teagasc, Athenry, Co. Galway Ireland; 8 Department of Agriculture, Food and the Marine, Sligo Regional Veterinary Laboratory, Sligo, Ireland; 9 School of Biology and Environmental Science (Emeritus Professor), University College Dublin, Belfield, Ireland

**Keywords:** Ticks, Tick-borne diseases, *Ixodes ricinus*, *Borrelia*, *Anaplasma*, *Babesia*, Louping ill

## Abstract

Throughout Europe interest in tick-borne agents is increasing, particularly with regard to those that can cause human disease. The reason for this is the apparent rise in the incidence of many tick-borne diseases (TBD’s). While there has never been a national survey of ticks or TBD’s in Ireland, the trend here appears to be the reverse with a decline in the incidence of some agents seemingly associated with decreasing tick numbers particularly on agricultural land. In the absence of robust baseline data, however, this development cannot be confirmed.

This review collates the limited information available from several dated published records on tick species and a small number of studies focused on certain TBD’s. Some pilot data on tick density and TBD agents collected in 2016 are also presented. The aim is to explore the particular situation in Ireland with regard to ticks and TBD’s and to provide a reference for future workers in the field.

## Background

Ticks are rarely considered serious pathogens in their own right. The bite itself usually causes little irritation although the lesion may become infected with *Staphylococcus aureus*, causing tick pyaemia and/or blow fly larvae, resulting in myiasis. Heavy infestations, particularly with adult female ticks, may cause anaemia [1]. Tick paralysis caused by salivary neurotoxins is mostly associated with certain African, North American and Australian tick species and affects humans, large ungulates, sometimes dogs and rarely cats [1–3]. Tick anaphylaxis, endemic in Australia, has rarely been reported elsewhere [4]. Moreover a peculiar correlation between tick bite and mammalian meat allergy in humans has been described recently mainly in the USA, but also several European countries and some other locations [4]. By far the greatest health impact of ticks, however, is related to their ability to transmit a variety of pathogenic micro-organisms which can cause disease in both humans and livestock [5].

## Main text

### Tick species present in Ireland and their host ranges

Whether a tick has the potential to serve as an important vector of disease depends not only on which pathogens it carries but also on the type of host it is likely to feed on. In Ireland, the most common tick species by far is the castor bean tick, *Ixodes ricinus*, which readily parasitizes reptilian, avian and a large range of mammalian hosts [6, 7]. For this reason *I. ricinus* is considered the most important tick-borne disease (TBD) vector, potentially transmitting TBD agents between wildlife, humans, livestock and pets. Another endemic species which also has a broad host range is the hedgehog tick, *Ixodes hexagonus* [8]. It infects cattle, sheep, horses, donkeys, cats and dogs as well as humans and diverse wild carnivores, rodents and birds. In many parts of western Europe *I. hexagonus* is second in abundance only to *I. ricinus* and acts as an important vector of zoonotic TBD’s [8], but its status as a disease vector in Ireland is not known [7]. *Ixodes canisuga*, the dog tick, which occasionally also feeds on sheep, horses and deer in addition to a range of wild carnivores, is also present. It is not known to transmit any significant pathogens [9]. A survey in the British Isles found that *I. ricinus* was the dominant tick species infesting dogs, particularly those that had visited woodland and bogland, *I. hexagonus* was the most common tick detected on cats [10], while infestation with *I. canisuga* was strongly associated with exposure to kennels and catteries. According to Martyn’s review [6] there was a single report of *Rhipicephalus sanguineus* in Belfast. Though no further details are provided, it is likely that this report is based on an imported case.

The remaining tick species that have been recorded here are primarily wildlife parasites and include *Ixodes trianguliceps* (which infests a range of rodents particularly wood mice and pygmy shrews and, rarely, horses and donkeys), *Ixodes verspertilionis* and *Argas vespertilionis* (bat parasites), and *Ixodes frontalis, Ixodes lividus, Ixodes rothschildi, Ixodes unicavatus, Ixodes uriae* and *Ornithodoros maritimus* (ticks of diverse native and migratory birds) [6, 11]. Of these only *I. trianguliceps*, A. *vespertilionis* and *I. uriae* have very occasionally been reported to feed on humans [6, 8].

### Tick habits and life cycles

With the exception of the soft ticks, *A. vespertilionis* and *O. maritimus,* all ticks reported from Ireland are ixodid, or ‘hard ticks’, i.e. their dorsal surface is covered in whole or in part by a chitinous plate-like shield, the scutum. Ixodid life cycles involves four stages, egg, six-legged larva, eight-legged nymph and adult [12]. Because each life cycle stage feeds only once, a disease agent must be able to persist through at least one moult or be passed to the eggs via the female’s ovary. In the former case the pathogen is acquired from a host by one stage e.g. larva and transmitted to another host by the following stage e.g. nymph. This is transstadial transmission. Some pathogens can persist through successive moults so that both nymphs and adults may be infective. Larvae are only infective if the pathogen infects the eggs. This is transovarial transmission. For a few pathogens both transstadial and transovarial transmission may occur.

Between feeds the microorganisms typically disseminate throughout the tick’s organs and multiply usually without affecting the fitness of the tick. Engorgement can take considerable amounts of time, e.g. in five ixodid species, feeding periods of 2 to 8 days have been reported for larvae, 4 to 10 days for nymphs and up to 13 days for adult females (adult males do not engorge and in *Ixodes* spp only feed briefly if at all) [12]. During these extended feeding periods saliva containing anticoagulants, cytolysins, vasoactive mediators and histamine blocking agents as well as excess fluid are injected into the feeding lesion facilitating the transmission of disease agents. Incidentally the host’s immune response to tick saliva components is thought to be a major determinant in tick host specificity [13]. Most ixodid ticks including *I. ricinus*, *I. hexagonus* and *I. canisuga* are ‘3 host ticks’, i.e. each life cycle stage feeds on a different host individual, frequently of a different species. Unfed ticks are highly responsive to carbon dioxide, body heat and odours, ammonia and physical disturbance and use these stimuli to locate their hosts.

*I. canisuga* and rodent and bird ticks such as *I. trianguliceps*, *I. frontalis* and *I. lividus*, are nidicolous, i.e. they remain in (endophilic) or adjacent to their hosts’ burrows and nests throughout their lives [12]. Here they are less exposed to unfavourable environmental conditions and the likelihood of encountering a host is high. In contrast, exophilic (non-nidiculous) species such as *I. ricinus* live freely in the environment and actively seek hosts [12]. They do this by positioning themselves on the tips of grasses and shrubs where they await passing animals with the front legs outstretched, a behaviour known as ‘questing’. It is primarily these questing ticks that can be collected by ‘blanket dragging’, a technique that involves drawing a white flannel cloth over the vegetation [8]. The cloth simulates a passing host and questing ticks cling to it. In between feeds exophilic ticks spend long periods of time deep within the cover of vegetation seeking shelter from adverse conditions. While *I. hexagonus* chiefly inhabits the sheltered habitat of their hosts’ nests [14], Ogden et al. [10] suggested that infestation patterns on cats and dogs indicated its life style was not strictly endophilic and these ticks might be better described as ‘harbourage nidicoles’ [12].

All tick species have temperature thresholds above which they become active. Interestingly recent research indicates that the thresholds for *I. ricinus* are adaptable depending on climatic conditions at the ticks’ original site, i.e. ticks from cooler climates become active at lower temperatures than those from warmer climates [15]. In addition to temperature, ixodid ticks are also sensitive to desiccation although the extent to which they are affected by humidity levels in the environment varies. *I. ricinus* for example requires a relative humidity of over 80% [16] while *I. canisuga* can survive under much drier conditions, a fact that is reflected in its prevalence in dog kennels [8]. In the short term, ticks can obtain water from subsaturated air by secreting hygroscopic fluid onto external mouthparts and re-ingesting the water-enriched fluid [17], however, continued survival of *I. ricinus* is only possible in micro-habitats of consistently high humidity.

In continental Europe tick activity typically follows a bimodal pattern with a major peak in spring and early summer and a second, lesser peak in autumn. In contrast, the mild and humid climate prevalent in Ireland allows tick activity to continue throughout the summer into autumn [18]. During the winter months ticks enter diapause, a hormonally-controlled decrease in activity and development [19]. As was described for threshold temperatures, the threshold photoperiod that stimulates diapause varies with the origin of the tick population, with ticks from higher latitudes differing from those from lower latitudes by several hours (reviewed by Gray et al. [19]).

Following mating, which in Ireland appears to occur chiefly off the host [20], female *I. ricinus* ticks lay hundreds of eggs in a single mass and die. The number of eggs is directly related to the size of their last blood meal [8]. Egg hatching may occur as early as two weeks following oviposition or may be delayed by up to one year [21]. Overall the tick life cycle can take between one and six years [8, 19], i.e. for some short-lived host species, ticks may outlive their hosts.

The only soft tick or argasid species reported from Ireland are *A. vespertilionis* and *O. maritimus.* Soft ticks lack a scutum, *Argas* spp have a granular, *Ornithodoros* spp a leathery surface. There are several nymphal stages in the argasid life cycle and adults feed repeatedly though only for short periods of time. Generally soft ticks are better adapted than hard ticks to withstand desiccation. Although some important diseases such as the tick-borne relapsing fevers and African swine fever are transmitted by soft ticks, world-wide there are much fewer species than ixodids and they are less economically important. *A. vespertilionis* and *O. maritimus* are nidicolous species infesting their hosts’ roosts or nests [8] and are vectors of various viruses of unknown significance [22].

### Distribution

For both, nidiculous and exophilic ticks, host availability is an important factor in determining distribution. It is thought that even in the absence of wildlife hosts, domestic animals such as sheep and cattle can maintain *I. ricinus* populations. In the wild, the tick feeds readily on cervid, other ruminant, rodent and avian hosts. Among these, deer who serve as hosts for larvae and nymphs as well as providing meeting and mating sites for sexually mature ticks [23, 24] are probably of primary importance, as several studies have reported positive correlations between deer and tick densities [23, 25]. Of the deer species that occur in Ireland, fallow and red deer are probably equally susceptible to ticks. However, because fallow deer are the most widespread and abundant deer species [26] and frequent open meadow, agricultural land, old- field habitats and so-called ecotones [27], transition zones between habitat types, such as the forest margin and the edges of paths or roads where tick densities are particularly high [28, 29], they are probably the most important wild tick host on the island. Tick infestation rates of sika deer indicate that they may be less susceptible to ticks (J. Gray, pers. observations).

As exophilic ticks spend at least 90% of their lifetime off the host in the environment, their distribution is also greatly affected by physical conditions in the microhabitat. Due to their pronounced susceptibility to desiccation, *I. ricinus* ticks are mostly found in areas where the vegetation provides a dense moisture-retaining ground layer and less permeable soil types promoting a moist microhabitat [30]. In drier locations, woodland and forests usually represent the most suitable habitats. In Ireland *I. ricinus* is also prevalent in open hill- and rough grazing land [20].

### TBD’s in general

There is no doubt that in the UK and Ireland *I. ricinus* is the principle vector of tick-borne disease [31]. While the presence of certain preferred host species can boost overall numbers of ticks as discussed above, their effect on tick infection rates is often more complicated. Specifically it has been argued that some important tick hosts may have a diluting effect because if they are not competent reservoirs their presence may help to block the disease cycle. It should be noted, however, that this hypothesis is still being debated in the literature [32–34]. The life stage most responsible for pathogen transmission depends on the mode of transmission of the pathogen involved (transstadial or transovarial) and, since life stages have specific host predilections, on whether these host species are competent reservoirs.

To date a number of tick-borne diseases have been reported from Ireland including Louping ill, borreliosis, anaplasmosis and babesiosis. Here we review what is known about the occurrence of each of these in Ireland and conclude by discussing the results of our own pilot study.

### Louping ill

Louping ill (LI) is caused by a flavivirus thought to have evolved from a precursor of tick-borne encephalitis (TBE) virus approx. 800 years ago [35]. Interestingly TBE, endemic to most of Europe and eastern Asia, is absent from the British Isles. In contrast LI virus is endemic in the UK and Ireland but mostly absent from continental Europe, although a small number of LI-like viruses have been described from geographically defined foci in Norway, Greece, Spain and Turkey [8, 35]. LI, also known as ovine encephalomyelitis infects various livestock and wildlife species in addition to sheep including cattle, goats, pigs, horses, mountain hare and red grouse [35, 36]. Human infections are extremely rare and usually associated with occupational exposure to infected carcasses rather than tick bite. Of all these potential hosts, only sheep and grouse are thought to develop viraemias high enough to infect engorging larval and nymphal ticks [35].

The initial febrile condition associated with the viraemic stage frequently goes unnoticed by the farmer. Often it is not until the virus enters the central nervous system and starts to disseminate that overt clinical signs become apparent, including depression, panting, nibbling, muscle tremors, incoordination, circling, ataxia and recumbency. However infections that have reached this stage usually result in death. There is no treatment for LI, but a vaccine containing inactivated virus particles grown in tissue culture (Louping-Ill vaccine, MSD) is available to protect naïve stock.

While recently weaned lambs moved to tick-infested pastures are thought to be most susceptible to disease [35], animals growing up in endemic areas usually develop some level of immunity resulting in reduced mortality rates of 5 to 10% as compared to 60% in newly introduced stock [36]. Barrett and colleagues [37] who carried out a serosurvey of 199 sheep carcasses submitted to the Sligo Regional Veterinary Laboratory for routine postmortem examination, reported a seroprevalence of 8.5%, but clinical manifestations of LI (non-suppurative meningoencephalitis) in just three animals (1.5%). The vast majority of seropositive sheep identified in this study (88%) were over 24 months of age.

As already mentioned, red grouse are highly susceptible to LI virus, with an estimated mortality rate of 80%. It is thought that in this host ingestion of infected ticks during grooming may also be an important route of infection [35]. What role, if any, LI has had in the dramatic decline of red grouse in this country is unclear [38].

In addition to LI virus, several serogroups of bunyaviruses and orbiviruses have been isolated from hard and soft tick species (*I. uriae*, *I. rothschildi* and *Ornithodoros maritimus*) collected from a seabird colony on Great Saltee Island [22]. Their clinical significance remains unclear.

### Lyme borreliosis

Lyme borrelosis (LB), also referred to as Lyme disease, is caused by the spirochaete *Borrelia burgdorferi* sensu latu (s.l.). It is not only considered the most prevalent arthropod-borne human disease in Europe and the US [16], but its incidence appears to be on the increase [39], although it is unclear whether this is due to genuinely higher infection rates or improved medical awareness and diagnostics. The symptoms of LB are non-specific and often mimic other diseases. Erythema migrans which develops at the site of the bite or elsewhere is the most common clinical sign (observed in about 89% of cases) [39] and usually resolves within a number of weeks even without antibiotic treatment. However, dissemination of the pathogen to other organs and tissues can give rise to a variety of symptoms including neurological manifestations (3%), Lyme arthritis (5%), borrelial lymphocytoma (painless bluish red nodules on ear, nipple or scrotum) (2%), acrodermatitis chronica atrophicans (long-standing bluish red atrophic lesions on extremities) (1%) and, rarely, cardiac manifestations (<1%) [39].

Of all domestic animals, dogs appear to be the most severely affected although reported cases are chiefly restricted to the US. The spectrum of disease is less complex than in humans with affected animals showing acute, recurrent lameness, occasionally with swollen, painful joints, fever and anorexia. Renal or cardiac involvement has also been described [40, 41]. While clinical signs such as lethargy, low-grade fever, lameness, uveitis, nephritis, hepatitis and encephalitis have been attributed to LB in horses [42], they are very rarely observed [40]. There is no unequivocal evidence that cats, small and large domestic ruminants are susceptible to disease although serosurveys in endemic areas suggest that they are frequently exposed to the pathogen.

At least 20 species are recognized within the *B. burgdorferi* s.l. species complex [43], only five of which are apparently associated with disease; *B. burgdorferi* sensu stricto (s.s.), *Borrelia afzelii*, *Borrelia garinii*, *Borrelia spielmanii* and *Borrelia bavariensis*. Three further species, *Borrelia bissettii*, *Borrelia lusitaniae*, and *Borrelia valaisiana* have occasionally been detected in patients, but are not recognized as important pathogens [39]. The different species vary slightly in their disease manifestations with *B. burgdorferi* s.s being mostly associated with arthritis, *B. afzelii* with degenerative skin conditions and *B. garinii* with neuroborreliosis though there is a considerable amount of overlap [16].

The human incidence of LB in Ireland is estimated at about 50 to 100 cases per year or a crude incidence of 0.6 per 100,000 [44, 45] with the west of the country being mostly affected. Neuroborreliosis which has been notifiable since 2012, accounts for between 8 and 18 cases annually. However, a review of acute LB cases carried out by Elamin et al. [46] revealed neurological involvement in 50% of cases, indicating that neuroborreliosis is probably underreported. To our knowledge the only surveys of *Borrelia* spp present in ticks ever to be carried out in Ireland date back to the 1990’s. Kirstein and colleagues [47, 48] reported infection rates of *B. burgdorferi* s.l. in questing ticks collected in a number of forests and national parks of between 3.5 and 26.7% (including 3.5% in Glenveagh and 18.8% in Killarney National park; 26.7% in Avondale, 4.4% in Lough Key and 16.4% in Portumna Forest Parks and 24.2% in Garinish Island). Molecular typing revealed the apparently non-pathogenic species, *B. valaisiana* to be the most common and widespread species (34.6%), followed by *B. garinii* (24.3%), *B. burgdorferi* s.s. (18.4%) and *B. afzelii* (6.6%). In 13.2% of infected ticks several spirochaete species were present.

Many attempts have been made to identify the most important reservoir hosts for *Borrelia* spirochaetes in the environment. Large mammals such as red, fallow and sika deer, cattle and sheep are certainly important reproductive hosts for ticks, and by feeding large numbers of all life cycle stages, their presence invariably serves to significantly boost tick numbers [23, 25]. However, because they are not competent hosts for the spirochaete, their contribution consists largely of uninfected ticks and, if anything, results in diluting the infection pressure [16, 23, 29]. While it has been reported that ticks can directly transmit the spirochaetes to each other while co-feeding on a host that is not susceptible [49], this transmission route is unlikely to be very important in nature.

In continental Europe, wood mice (*Apodemus* spp.) and bank voles (*Myodes glareolus* are considered important components of the transmission cycle of *B. burgdorferi* s.l. [50]. However, although wood mice are common in Ireland and bank voles have been spreading from their initial introduction site in Ireland in the 1920’s [51], studies in Killarney National Park [29, 52] and Connemara National Park [53] suggested that rodents are of limited significance as reservoir hosts in this island. On the other hand wild birds, particularly woodland species such as pheasants and blackbirds, may have a significant role [16], a hypothesis that is further supported by the relative rarity throughout the country of the rodent-associated genospecies *B. afzelii,* but the common occurrence of bird-associated genospecies such as *B. valaisiana* and *B. garinii* [47, 48]*.* In some habitats, medium sized wildlife species such as squirrels, and hedgehogs may also serve as reservoir hosts. It is generally agreed that the greatest risk from LB is associated with deciduous woodlands that have high tick densities and a diverse mix of host and *Borrelia* species [16].

There is no doubt that *I. ricinus* is the chief vector of the spirochaete in Ireland, but other species such as *I. hexagonus* and the rodent tick, *I. trianguliceps* may also contribute to its circulation in their wildlife host populations [16]. The seabird tick, *I. uriae* is a recognized vector of *B. garinii* [54] but although it readily feeds on humans, human exposure to this endophilic species is probably a relatively rare event. As each feed increases the chance of exposure, infection rates in *I. ricinus* usually increase with progressive instars [23, 47], however, due to their relative abundance nymphal ticks are more important vectors than adults. On the other hand, because transovarial transmission of *Borrelia* spp is rare or absent the risk associated with larval ticks is considered negligible [16, 39]. As engorgement commences, dormant spirochaetes in the midgut are stimulated to multiply and develop into infective stages [39]. Because this process takes several hours (the exact period is under debate and ranges from 4 to 48 h) infections can be avoided by prompt removal of the tick [8, 16, 39].

### Anaplasmosis

While LB is the most common tick-borne human disease, *A. phagocytophilum* is the most prevalent tick-transmitted animal pathogen [55]. The epidemiology of this gram-negative Rickettsial organism is complicated by the fact that the species was only created recently by Dumler et al. [56] who merged all species that infect granulocytes, i.e. *Ehrlichia phagocytophila*, *Ehrlichia equi* and the agent of human granulocytic anaplasmosis into a single entity called *Anaplasma* (*Ehrlichia*) *phagocytophila* comb. nov. subsequently renamed *A. phagocytophilum* [57]. The authors justified this amalgamation on the basis of high sequence homology in the 16S rRNA gene (99.1%) and several other gene loci. However, the strains within the species display significant biological differences with regard to host specificity and distribution [55, 58]. For example, only certain strains seem to be infectious to humans. In the USA human granulocytic anaplasmosis is now considered a significant emerging zoonosis while in Europe fewer than 100 cases have been reported in total [58]. On the other hand, ovine and bovine infections have only ever been diagnosed in Europe while cases in horses, cats and dogs occur in both the US and Europe. In Ireland, there are no records of human, canine, feline or equine granulocytic anaplasmosis. To our knowledge the only strains or genotypes present are those that infect wild and domestic ruminants [59, 60] although it is possible that other, wildlife strains are also being maintained in rodent, bird or small wild mammal populations (such as hedgehogs). However these wildlife cycles are probably independent from those in domestic animals and humans, involving chiefly endophilic wildlife vectors such *I. hexagonus*, *I. trianguliceps* and certain bird-feeding ticks [58].

In sheep, cattle and goats, *A. phagocytophilum* infections, commonly referred to as tick-borne fever (TBF), are characterized by fever, weakness, anorexia, and occasionally, respiratory distress. Once these factors interfere with the ability of young lambs to maintain contact with the dam high rates of morbidity and mortality are observed especially on rough upland pasture. Mature animals, naïve cows and ewes newly introduced into endemic areas are more likely to abort, while males may be temporarily sterile, possibly as a consequence of pyrexia [61, 62]. In dairy cows the most notable clinical sign is a significant, sudden drop in milk yield [14].

In addition to these direct effects of TBF, infection of granulocytes also results in generalized immunosuppression predisposing animals to other infectious and tick-borne diseases particularly tick pyaemia, LI, pneumonic pasteurellosis and listeriosis [36, 63]. In fact tick pyaemia, caused by coinfection of the tick bite with *S. aureus*, is the most common and serious complication of ovine granulocytic anaplasmosis in the UK causing approx. 300,000 cases of lamb tick pyaemia annually [58]. Similarly animals that already suffer the clinical effects of TBF generally show more severe signs in response to subsequent *Babesia* infections [64] which are less responsive to treatment [65]. Interestingly though, during synchronous infections with the two pathogens presence of the Rickettsial organisms appears to suppress the protozoal agent [66], indicating that the initial stages of babesiosis may be partially immune-mediated.

With regard to its development in the tick host, *A. phagocytophilum* shares some characteristics with LB. For example the organism is transmitted transtadially, and transmission to a new vertebrate host does not occur immediately after attachment but requires several hours of development and multiplication in the tick’s digestive tract and salivary glands. Direct transmission between co-feeding ticks has been reported but appears to be rare [58].

### Babesiosis

Many of the most important and pathogenic *Babesia* species of domestic animals such as *Babesia bovis*, *Babesia bigemina*, *Babesia canis*, *Babesia gibsoni* and *Babesia caballi*, are transmitted by ticks thought to be absent from Ireland [6–8, 14]. Therefore any unusual cases that are observed here should be investigated closely to ascertain whether they are associated with recent travel to endemic areas. *I. ricinus* serves as vector for the cattle parasite, *Babesia divergens*, by far the most important species in Ireland. In addition *I. ricinus* also transmits several cervine *Babesia* species that are not known to be of clinical significance in either wildlife, livestock or indeed, humans (see below). Finally *I. ricinus* can transmit, *Babesia ovis,* which to our knowledge, has never been reported in Ireland.

*B. divergens* is a single-celled Apicomplexan parasite that infects erythrocytes causing fever, anemia, anorexia, tachypnea and tachycardia (reviewed by Zintl et al. [67]). Mildly affected animals usually have low levels of parasitaemia and make an uneventful recovery. Severe cases however, are characterized by parasitaemias of 30 to 45% (and over), extensive erythrocyte destruction, jaundice, severe dehydration, hemoglobinuria and pipe-stem diarrhoea. Once body temperature falls to near or below normal and diarrhea is replaced by constipation, animals are usually moribund. In 1983, case fatality rate was estimated at around 10% [68]. More recent figures are unfortunately not available.

The epidemiology of bovine babesiosis is unusual in that calves less than 9 to 12 months of age are resistant to disease, yet fully susceptible to infection, a phenomenon known as ‘inverse age resistance’ [69]. In areas of high infection pressure this is thought to result in ‘endemic stability’ as most or all animals are infected in their first year of life while they are still protected from disease. Under those circumstances clinical disease is chiefly observed in naïve adult cattle that have been moved into the area. In the past, *B. divergens* was a very common and important parasite in Ireland particularly in parts of the west, northwest and Shannon catchment area [68]. In fact it was considered such a serious constraint to the cattle industry that a live vaccine was developed and successfully deployed during a pilot vaccination programme [70]. However, several questionnaire surveys carried out over the last three decades indicate that the incidence of bovine babesiosis has declined dramatically from an estimated 1.7% in the 1980’s to around 0.06% in 2013 [71].This decline has been attributed to a number of factors that may have caused a decrease in tick densities such as the clearing and ‘improvement’ of pastures that were formerly prone to tick infestation and their deployment for different livestock or crop. Although there is no scientific evidence, there is a widely held belief among farmers and veterinarians that the widespread use of macrocyclic lactones may also have had an indirect effect on the level of tick infestation on pasture.

In addition to cattle, *B. divergens* can also infect humans, and although with less than 50 records in the whole of Europe to date, these cases are extremely rare they are potentially life-threatening [72–74]. Until recently it was thought that zoonotic infections were restricted to asplenic patients, however, a small number of recent cases have been reported in spleen-intact, immunocompetent individuals [73, 75]. To our knowledge four Irish cases (3 in asplenic patients) have been described to date, the most recent of which occurred in 2015 in an elderly Irish farmer who was hyposplenic possibly as a result of adult coeliac disease [76]. The rodent species *Babesia microti* which causes more than a thousand human cases in the USA annually, is not an important zoonotic agent in Europe. Even though *B. microti* is frequently detected in small mammals, autochthonous clinical cases in Europe are extremely rare. To date a single severe case in an immunocompromised patient [77] and a small number of mild flu-like infections characterized by non-specific flu-like symptoms in immunocompetent individuals have been reported [78, 79]. *Babesia venatorum* another emerging human pathogen in Europe appears to be chiefly associated with roe deer [80]. This deer species is absent from Ireland, and so presumably is its parasite [81].

In contrast to the two bacterial organisms, *Borrelia* and *Anaplasma*, ticks appear to acquire infections with *B. divergens* chiefly as adult females that, following engorgement transmit the parasite transovarially [82]. Once established in the tick host, the protozoan can apparently persist in *I. ricinus* as far as the second generation larvae even if the intervening tick stages feed exclusively on uninfected blood. As a result it has been hypothesized that in the field, infections may be maintained in tick populations for at least 4 years even in the absence of an infected bovine host [69]. As described for LB and anaplasmosis, inoculation of infective sporozoites occurs during the latter half of the blood meal [82], i.e. risk of infection can be reduced significantly by prompt removal of the tick.

### Pilot study

**Fig. 1:**
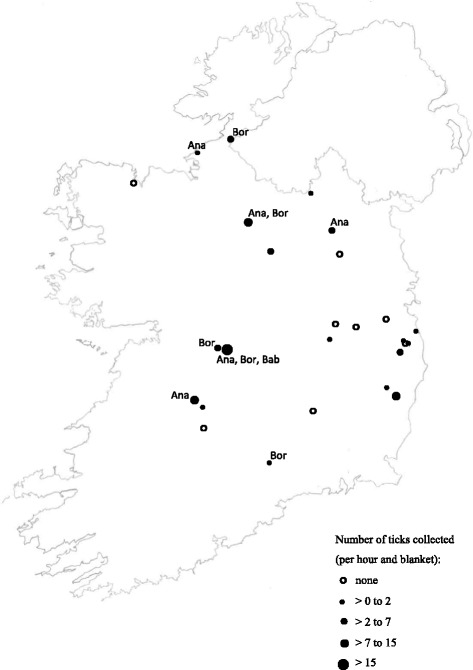
Tick abundance and presence of tick-borne disease agents as indicated by the pilot survey carried out in 2016. Only ticks collected by blanket dragging are included. Ana, Bor, Bab indicate presence of *A. phagocytophilum*, *Borrelia & Babesia* spp in pooled tick samples

While no comprehensive survey of ticks has ever been carried out in Ireland, inferences on the distribution of *I. ricinus* have been made based on the occurrence of bovine babesiosis and LB. According to several questionnaire surveys and confirmed clinical cases, both diseases are most prevalent along the west and north west of Ireland and the Shannon river system [44, 71]. Anecdotally there are also foci in County Wicklow. A pilot tick survey carried out by us in 2016 roughly confirmed these suppositions (Fig. 1). Between April and July 2016 we collected 151 ticks (8 larvae, 122 nymphs and 21 adults) by blanket dragging [8] in 26 sites from 17 counties. These included 13 sites on farmland where owners had reported issues with tick-borne diseases, seven woodland and forest sites, four sites in bogland and marsh and two in rough scrub. The site with the highest tick density by far was Portumna Forest Park, County Galway where 116 ticks were collected per hour and blanket. This compares to just 1.2 ticks per hour and blanket on average in the other woodland sites. In farm, marsh/bog and scrub sites average numbers of ticks collected per hour and blanket were 3.2, 1.8 and 0.8 respectively. It is important to stress that these data are preliminary as sampling was carried out by a number of different operators, often under suboptimal weather conditions. In addition there was poor coverage of the west of the country and sites were not selected at random and only visited once. Sampling of a larger number of sites, stratified by vegetation type and land use and using standardized methods will be necessary to draw an accurate distribution map of *I. ricinus* in Ireland.

In addition to the ticks collected by blanket dragging, 28 ticks (11 larvae, 14 nymphs and 3 adults) were collected from wood mice trapped in a woodland in County Kildare and 18 (3 nymphs and 15 adults) from bovine (*n* = 7), ovine (*n* = 6), feline (*n* = 4) and human hosts (*n* = 1) collected by the Regional Veterinary Laboratory, Sligo. As expected all ticks collected by blanket dragging were identified as *I. ricinus*. Blanket dragging is selective for the collection of exophilic ticks and *I. ricinus* is the only known exophilic species present in Ireland. With the exception of the three adult ticks collected from wood mice which were identified as *I. trianguliceps* all other ticks found attached on their hosts were identified as *I. ricinus*.

All ticks were screened for the presence of known bacterial and protozoal TBD agents using PCR-based methods as described by Michelet et al. [83]. Engorged ticks collected from hosts were analysed individually, while flagged ticks were pooled (by sample site and life cycle stage) in groups of two to five ticks.

PCR analysis of flagged ticks revealed the presence of *A. phagocytophilum* in five sampling sites (Fig. 1), two of which were farm and marsh/bog sites respectively and one a forest (Portumna Forest Park). Similarly *Borrelia* spp were detected in five sites, three of which were farm sites, one a marsh/bog and one in Portumna Forest Park. In contrast *Babesia* was only detected in two pools of ticks collected in Portumna Forest Park. Our results did not indicate any correlation between TBD agents and tick densities or land use/vegetation type.

Among ticks collected directly from hosts, *A. phagocytophilum* was by far the most common TBD agent (detected in 67% of ticks collected from sheep, 100% of ticks from cats, 83% of ticks from cattle, 20% of ticks from wood mice and in the single tick collected from a human host). *Borrelia* spp were only present in one tick collected from cattle and three ticks (one *I. ricinus* and two *I. trianguliceps*) from wood mice. *Babesia* spp were detected in a single tick that had engorged on a bovine host. When considering these data it is important to bear in mind that any TBD agent detected may have originated either from the tick or the host it was collected from.

All *Borrelia* spp detected during this study were identified as *B. garinii*, *B. afzelii* or *B. valaisiana*.

All samples were negative for the following TBD agents: *Ehrlichia* spp, *Rickettsia* spp, *Bartonella* spp, *Francisella tularensis*, *Coxiella burnettii* and *Theileria* spp. For logistical reasons ticks were not screened for the presence of LI virus or any other viral disease.

## Conclusions

Our pilot survey confirmed that ticks in Ireland carry a limited diversity of TBD agents. The comparatively high prevalence of *A. phagocytophilum* is supported by anecdotal information from farmers and veterinarians who have been highlighting the clinical importance of this agent for some time, stressing that it should be considered as a differential in milk drop syndrome, accompanied by unexplained fever and respiratory signs in early summer. In addition its effect on other concurrent endemic livestock diseases must be considered. While a small number of commercial laboratories offer immunodiagnostic screens for the presence of *A. phagocytophilum* antibody, there are currently no published prevalence data in either cattle or sheep. It is important to stress, however, that the strain (s) present in Ireland appear to be specific to ruminants and are not likely to represent a public health risk.

The dearth of information on ticks and TBD’s in Ireland contrasts with the considerable amount of resources dedicated to vector-borne disease surveillance in many parts of Europe. Significantly the trend in declining tick numbers (at least on agricultural land) as indicated by the decreasing incidence of bovine babesiosis runs contrary to what is being observed throughout Europe where many tick species and TBD’s are reported to be increasing, both in numbers and geographic range [84, 85]. It is therefore not inconceivable that the current trend in Ireland may reverse, particularly since the reasons for the apparent decline are not well understood. Furthermore in the absence of regular tick collection and screening, changes in TBD agents will not be detected until clinical cases are observed in the field, at which point the pathogens are likely to be established in the tick and host reservoir populations, rendering effective control measures more difficult and costly.

## Abbreviations

LB: Lime borreliosis
LI: Louping ill
TBD: Tick-borne disease
